# Putative Mitoviruses without In-Frame UGA(W) Codons: Evolutionary Implications

**DOI:** 10.3390/v15020340

**Published:** 2023-01-25

**Authors:** Andrés Gustavo Jacquat, Martín Gustavo Theumer, José Sebastián Dambolena

**Affiliations:** 1 Facultad de Ciencias Exactas Físicas y Naturales (FCEFyN), Universidad Nacional de Córdoba (UNC), Córdoba 5000, Argentina; 2 Instituto Multidisciplinario de Biología Vegetal (IMBIV), Consejo Nacional de Investigaciones Científicas y Técnicas (CONICET), Avenida Vélez Sarsfield 1611, Córdoba 5016, Argentina; 3 Departamento de Bioquímica Clínica, Facultad de Ciencias Químicas (FCQ), Universidad Nacional de Córdoba (UNC), Córdoba 5000, Argentina; 4 Centro de Investigaciones en Bioquímica Clínica e Inmunología (CIBICI), Consejo Nacional de Investigaciones Científicas y Técnicas (CONICET), Córdoba 5000, Argentina

**Keywords:** public databases, narna-levi, *Mitoviridae*, *Kvaramitovirus*, Arkeomitovirinae, Mitovirinae, UGA codon, UGG codon, origin, evolution, LECA

## Abstract

Mitoviruses are small vertically transmitted RNA viruses found in fungi, plants and animals. Taxonomically, a total of 105 species and 4 genera have been formally recognized by ICTV, and recently, 18 new putative species have been included in a new proposed genus. Transcriptomic and metatranscriptomic studies are a major source of countless new virus-like sequences that are continually being added to open databases and these may be good sources for identifying new putative mitoviruses. The search for mitovirus-like sequences in the NCBI databases resulted in the discovery of more than one hundred new putative mitoviruses, with important implications for taxonomy and also for the evolutionary scenario. Here, we propose the inclusion of four new putative members to the genus *Kvaramitovirus*, and the existence of a new large basally divergent lineage composed of 144 members that lack internal UGA codons (subfamily “Arkeomitovirinae”), a feature not shared by the vast majority of mitoviruses. Finally, a taxonomic categorization proposal and a detailed description of the evolutionary history of mitoviruses were carried out. This in silico study supports the hypothesis of the existence of a basally divergent lineage that could have had an impact on the early evolutionary history of mitoviruses.

## 1. Introduction

Viruses belonging to the family *Mitoviridae* have a small (2151–4955 nt) non-segmented positive-sense single-stranded (+ss) RNA genome which codes for a single protein, namely, the RNA-dependent RNA polymerase (RdRp) [1,2]. These non-virion-forming viruses do not have an extracellular phase, as they are vertically transmitted RNA elements. Although it is likely that they can migrate to the cytoplasm of host cells [3,4], their replicative cycle occurs within the mitochondrial matrix of their hosts [1,5,6,7,8]. Mitovirus sequences have been found in the genome (nuclear and/or mitochondrial) of some eukaryotic organisms, especially in embryophytes [6,9,10], indicating their ability to endogenize in their plant hosts. However, the search for mitovirus sequences within the genome of other taxa has been unsuccessful [11,12,13,14,15]. To date, mitoviruses have been found to replicate in fungi [1,16], embryophytes [6,7] and animals [8]. Those that replicate in fungal and animal mitochondria have a similar UGA/UGG codon ratio to that of host core mitochondrial genes [5,8], while mitoviruses that replicate in plant mitochondria do not have internal UGA codons, since this codon encodes a translation stop signal in plastids [6,7]. The UGA and UGG codons are two synonymous codons for the amino acid (aa) tryptophan (W) in some genetic codes, such as the mitochondrial genetic codes of invertebrates and fungi. However, UGA is a translation termination signal for the standard genetic code, while only the UGG codon encodes for W [17].

Mitoviruses and narnaviruses (family *Narnaviridae*), which are other vertically transmitted naked RNA cytoplasmatic elements, are considered to be the simplest RNA viruses [2]. According to phylogenetic reconstructions, members of both these taxonomic families form a monophyletic lineage at the tree root of the global RNA virome. Moreover, mitoviruses and narnaviruses share a recent common ancestor with +ssRNA bacteriophages (class *Leviviricota*), forming a basal evolutionary lineage of the global RNA virome named “Narna-Levi” [18], “Branch 1” [2] or “phage-related (+)RNA viruses” [19]. Taxonomically, a total of 105 species and 4 genera (*Unuamitovirus*, *Duamitovirus*, *Triamitovirus* and *Kvaramitovirus*) of mitoviruses have been recognized by the International Committee on Taxonomy of Viruses (ICTV) [20], and recently, a new mitovirus genus named “Kvinmitovirus” has also been proposed [21]. Nevertheless, the systematic classification of the mitoviruses remains under constant review.

RNA sequencing and metatranscriptomics studies are a major source of hundreds of new “Narna-Levi”-like sequences, and consequently, these new sequences are continually being added to open databases [18,22,23,24,25,26]. Moreover, RNA sequencing studies in organisms for purposes other than virus identification are a very valuable source for identifying new putative mitoviruses. For example, a clade of putative mitoviruses associated with animals was recently identified from public sequence databases [21]. In addition, although empirical evidence is still needed for its confirmation, the *in silico* approach based on similarities between protein sequences and close evolutionary relationships has revealed the existence of a new mitovirus lineage (clade “Kvinmitovirus”) [21]. This lineage was previously hidden from phylogenetic approaches based on hundreds or thousands of sequences obtained in metatranscriptomic studies. Following a similar approach to the one mentioned above, the aim of this article was to examine mitovirus-like sequences from different open databases to try to reveal new phylogenetic associations and to evaluate their potential contribution to the current knowledge about the evolutionary history of the family *Mitoviridae*.

## 2. Materials and Methods

### 2.1. Open Access Databases

In the present study, two public libraries of nucleotide (nt) and protein sequences were explored: the Transcriptome Shotgun Assembly (TSA) database and the non-redundant (nr) protein sequences database, both from the National Center for Biotechnology Information (NCBI, U.S. National Library of Medicine, Bethesda, MD, USA; accessed on 10 June 2022).

### 2.2. Bioinformatic Tools

Mitovirus-like sequences were scanned using the tBLASTn and BLASTp algorithms implemented by default at the web server of the NCBI Basic Local Alignment Search Tool (BLAST) [27] program: https://blast.ncbi.nlm.nih.gov/Blast.cgi (accessed on 15 September 2022).

Hypothetical coding regions, aa sequences and codon composition were predicted using ORFfinder (translation table numbers 1 and 4) on the web server https://www.ncbi.nlm.nih.gov/orffinder/ (accessed on 15 September 2022) and the Translate program [28]. The conserved protein sequence of Mitovirus RpRd (NCBI accession code: pfam05919: Mitovir_RNA_pol) was corroborated against CDD v. 3.20, using the NCBI CD-Search service [29], implemented by default at https://www.ncbi.nlm.nih.gov/Structure/cdd/wrpsb.cgi (accessed on 15 September 2022). A positive coincidence was considered for an e-value ≤ 1.00 × 10^−10^.

To estimate the overall percent of pairwise sequence identity for all deduced proteins included in the phylogenetic studies, the Sequence Demarcation Tool (SDT) v. 1 program [30] was used (the ClustalW algorithm was chosen to compute the identity score for each pair of sequences). For multiple sequence alignments, several programs were utilized: (i) CLUSTAL-Omega v. 1.2.4 [31] at the https://www.ebi.ac.uk/Tools/msa/ (accessed on 15 September 2022) web server, (ii) Multiple Alignment using Fast Fourier Transform (MAFFT) v. 7.505 [32] at https://mafft.cbrc.jp/alignment/server/index.html (accessed on 15 September 2022) [33], and (iii) Profile Multiple Alignment with Predicted Local Structure (PROMALS) [34] at the http://prodata.swmed.edu/promals/promals.php web server. Access to these servers was between October 1, 2022 and January 18, 2023.

Phylogenetic trees by the Maximum Likelihood method were generated with IQ-TREE v. 1.6.11 [35]. To determine the optimal aminoacidic substitution matrix (model of molecular evolution) according to the Bayesian Information Criterion, the ModelFinder program [36] was used. The statistical support of branches was estimated by the Ultrafast Bootstrap 2 (UFBoot2) [37] method. These three programs were run on the Los Alamos lab web server [38]. For mitoviruses (*Mitoviridae*) phylogenetic reconstructions, five members from the genus *Unuamitovirus*, five from the genus *Duamitovirus*, five from the genus *Triamitovirus*, one from the genus *Kvaramitovirus* and five from the clade “Kvinmitovirus” were arbitrarily included. Henceforth, throughout this article, we refer to these mitoviruses as “formal mitoviruses”, since they were formalized by the ICTV [20]. For simplicity, “formal mitoviruses” will include the members recently incorporated in the proposed new genus “Kvinmitovirus” [21]. To perform a deeper phylogenetic analysis, 25 members of the family *Narnaviridae*, 23 members of *Botourmiaviridae*, 20 members of “Narliviridae” and 20 members of the family “Leviviridae” (*Fiersviridae*) were included in this study. Details of all included sequences are given in Table S1. As an outgroup to rooted phylogenetic trees, the 20 RNA replicase sequences from “Leviviridae” family were chosen, taking into account the most complete and robust phylogenetic studies [2,19]. All ML-trees were displayed as generalized midpoint-rooted rectangular phylograms, using MEGA X v. 10.1.5 software [39].

## 3. Results and Discussion

### 3.1. Search for Putative Mitoviruses in the Open Databases

First of all, assembled contigs with mitovirus-like sequences were scanned on the NCBI TSA database using the tBLASTn mode of the NCBI BLAST web program. We used the tBLASTn strategy since its algorithm is more sensitive than BLASTn for matching putative mitoviral genomes with a high degree of divergence with respect to the query. In order to increase the sensibility for finding mitovirus domains in the tBLASTn search, we used an arbitrarily selected short query that only included the aa sequence of the conserved protein domain (CPD) of the mitovirus RdRps (highest evolutionary conserved region among known mitoviruses), which is composed of six conserved aa sequence motifs (I, II, III, IV, V and VI [40]). This query was the conserved region of the RNA replicase coded by Sclerotinia sclerotiorum mitovirus 3 (NCBI accession code: AGC24232.1; coordinates 265 aa–463 aa), the formal member of the viral species *Duamitovirus scsc 3*. Using this query, tBLASTn searches were performed on databases for different clades of eukaryotes (clade-limited tBLASTn search). These searches included all major taxa of eukaryotes: *Metazoan, CruMs, Malawimonadidae, Fornicata, Parabasalia, Heterolobosea, Euglenozoa, Rhodophyta, Glaucophyceae, Charophytes, Chlorophyta, Stramenopiles, Alveolata, Rhizaria, Haptista, Ancyromonadida and Cryptomonadida* (Table S2). In total, 247 TSA sequences were obtained, which were manually curated. Only those sequences with a hypothetical single open reading frame (ORF) of >1000 nt and with a hypothetical deduced protein (translation table #4 or #1) containing the Mitovirus RdRp CPD (NCBI accession conde pfam05919; e-value ≤ 1.00 × 10^−10^) were included in our study. In addition, to avoid the inclusion of redundant sequences, contigs with a very high sequence similarity (pairwise identity percentage greater than 90%) were eliminated, with only one of these being preserved. These sequences, with a high degree of similarity, were considered as putative strains of the same putative viral species or as truncated contigs generated from the same original transcript. These selection criteria for mitovirus-like TSA sequences were previously applied by Jacquat et al. [21].

**Figure 1:**
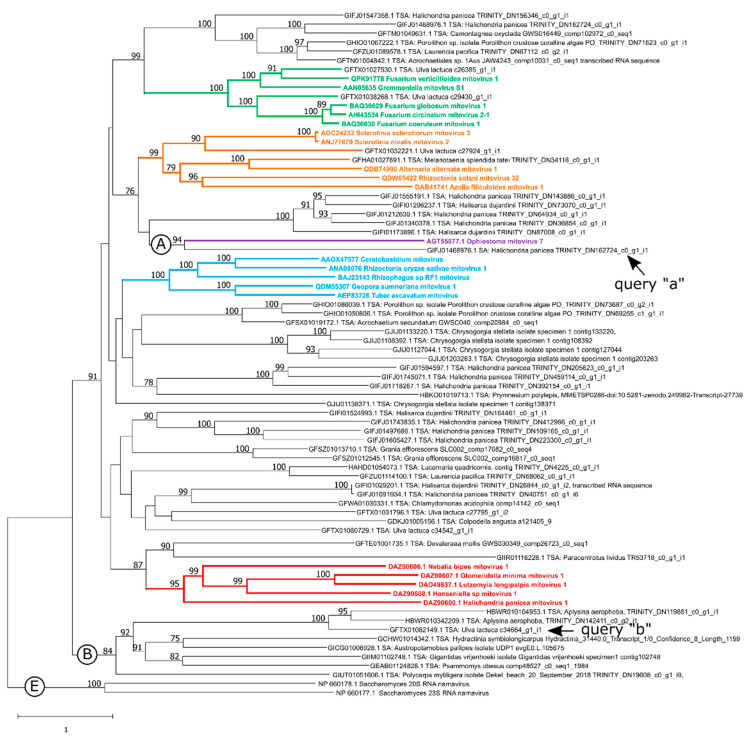
Generalized midpoint rooted tree of RNA replicase aa sequences. The deduced Mitovirus RdRps identified in the TSA database are labeled with the NCBI accession number, and the mitovirus formally accepted by the ICTV (colored branches) are labeled with the NCBI accession code and virus name. The color-coding of the branches: green, orange, cyan and purple indicates a member of the genus *Unuamitovirus, Duamitovirus, Triamitovirus* and *Kvaramitovirus*, respectively. Red branches indicate members of the recently proposed genus “kvinmitovirus”. Formal members are indicated in colored bold type. Clades discussed in the main text are indicated with a letter (“A” and “B”). Letter E indicates the external group (two *Narnaviridae* members). An arrow indicates the sequence used as the query to search for similar sequences deposited in open databases (details in the article). Sequence alignment: MAFFT (L-INS-i). Tree construction method: Maximum Likelihood. Evolutive model: LG+F+R6. Node support values are displayed as percentages (only values ≥ 75% are shown). The bar indicates one substitution (estimated median number) per alignment site.

After sequence curing, only 54 TSA sequences were considered to be putative near-complete mitovirus genomes. These were then subjected to an alignment with formal mitoviruses (see Materials and Methods section) using the MAFFT (strategy: L-INS-i) aligner. The obtained midpoint-rooted phylogram showed several highly-supported clusters (Figure 1). However, in order to simplify the analyses in the present article, we focused only on those that were the most taxonomically and phylogenetically relevant, according to some considerations described below. Surprisingly, the obtained results revealed an interesting clustering between Ophiostoma mitovirus 7 (OnuMV7), a unique member of the species *Kvaramitovirus opno7* belonging to the monospecific genus *Kvaramitovirus* and the TSA sequence under the NCBI accession code GIFJ01468976.1. Curiously, the last two revisions reported by the Mitoviridae Study Group (SG) [20] and Jacquat and colleagues [21] found great topological instabilities of OnuMV7 for all phylogenetic reconstructions, indicating the genus *Kvaramitovirus* to be a “lonely” and “small” lineage with no clear evolutionary relationships. Therefore, the clustering reported here could be revealing the existence of a new representative member of the genus *Kvaramitovirus*. This association was analyzed throughout the present article, and provisionally, this two-member clade was referred to as “Clade A”.

The above-mentioned ML-tree construction also showed another interesting clade that deserves to be explored in depth (Figure 1). This was composed of eight TSA sequences that branched at the base of the tree to form a robust basally divergent putative lineage. This clade was analyzed throughout this study, and provisionally, this was referred to as “Clade B”. This branching pattern could reflect the existence of a sister lineage to existing mitoviruses, with an early evolutionary origin in the history of the viral family. This hypothesis was studied in the present work (see below) to investigate the evolutionary stage of mitoviruses, especially in the earliest evolutionary stages of the family.

In order to tentatively expand the number of members in the obtained clades “A” and “B”, a search of putative mitoviruses for aa sequence similarity was performed using BLASTp software. Thus, we searched for aa sequences recorded at the NCBI nr Protein Sequence database. Regarding our attempt to expand “Clade A”, the deduced protein from the sequence recorded under the NCBI accession code GIFJ01468976.1 was used as the query (query “a”: Figure 1), with this query being the only putative virus to branch along with OnuMV7. Furthermore, a deduced protein that was clustered within clade “B” was arbitrarily chosen as the query to try to expand “Clade B”. This was the TSA sequence with the NCBI code GFTX01082149.1 (query “b”: Figure 1). The resulting first 250 hits of each BLASTp search were examined for sequences that were phylogenetically clustered within the preset clades “A” and “B”.

In addition to these searches, a data set of 8469 hypothetical proteins generated from 442 RNA sequencing libraries (NCBI BioProject accession code: PRJNA716119) reported by Sadiq et al. [41] and Chen et al. [42] were also inspected to be able to tentatively expand the number of members of the obtained clades “A” and “B”. These sequences were filtered to retain only hypothetical proteins with a Mitovirus RdRp CPD. This filtering enabled 574 deduced proteins to be identified as putative members of *Mitoviridae*. This set of 574 mitoviral proteins was then scanned and analyzed for members that evolutionarily fit within the clades “A” and “B”. Finally, the sequences obtained from the BLASTp searches and the bioproject PRJNA716119 that clustered within clades “A” and “B” were inspected to eliminate redundant sequences (pairwise alignments with an identity greater than 90%) and sequences lacking one protein motif (I–VI) of Mitovirus RdRp. The obtained results demonstrated that a total of four new putative mitovirus were clustered into “Clade A”, while 144 new putative mitovirus sequences were clustered into “Clade B”. All sequences are described in the supplementary Table S3.

### 3.2. New Putative Mitoviruses in the Phylogenetic Context of the Phylum Lenarviricota

**Figure 2:**
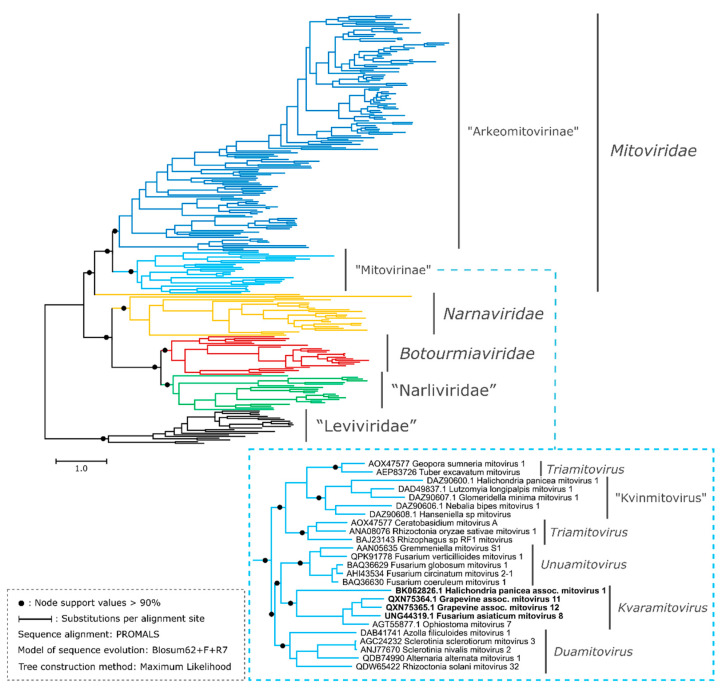
Generalized midpoint-rooted tree of 254 RdRp aa sequences encoded by representative members of the phylum *Lenarviricota*. Branches color-code: blue indicates the proposed subfamily “Arkeomitovirinae” (“Clade B”), cyan indicates formal mitoviruses fitted to the proposed subfamily “Mitovirinae” (formal genera *Unuamitovirus, Duamitovirus, Triamitovirus* and *Kvaramitovirus*, and also the proposed genus “Kvinmitovirus”). The family *Mitoviridae* comprises members of the blue and cyan branches. Orange, red and green indicate the families *Narnaviridae*, *Botourmiaviridae* and “Narliviridae”. New members proposed for the genus *Kvaramitovirus* (“Clade A”) are indicated in bold type in the sub-tree corresponding to the proposed “Mitovirinae” subfamily. The supplementary Figure S2 shows the tree displayed with the labels of the 254 members and the values of the nodes. The phylogenetic reconstructions are detailed in the Materials and Methods section.

A deep evolutionary reconstruction including members of the “Narna-Levi” clade (formally phylum *Lenarviricota*) was performed in order to assess the evolutionary relationships of the two new clades studied in the present work. It is worth mentioning that RNA replicases encoded by “Narna-Levi” viruses have a low overall similarity among the different taxonomic families which complicates the construction of a realistic tentative phylogenetic tree [41,43]. In particular, the aa sequences included in the present study (Table S1) showed a global pairwise alignment identity of 27.91% (SD: 9.58%), indicating a technical limitation [44]. To confront this difficulty, we conducted a phylogenetic reconstruction using the PROMALS aligner that generates a probabilistic consistency-based progressive multiple structure-sequence alignment [34]. The probabilistic consistency-based scoring combines both aa similarity information and secondary structure similarity information through the profile-profile comparison using the hidden Markov model. PROMALS offers certain advantages over traditional MSA algorithms for distantly related protein homologs since it was designed to align divergent sequences [34,45].The obtained phylogram is shown in Figure 2. To support further deductions based on the tree derived from PROMAL, we also evaluated other alignment methods to construct trees: CLUSTAL-O and MAFFT / FFT-NS-i (see Figure S1).

The obtained phylogenic tree showed three main clades whose phylogenetic relationships were consistent with a recent comprehensive phylogenetic study of the phylum *Lenarviricota* [41] (Figure 2). *Narnaviridae*, *Botourmiaviridae* and “Narliviridae” form a sister clade to *Mitoviridae*, with “Leviviridae” being a basal group. This three-way topology was also observed in the phylograms generated from the alignments with CLUSTAL-O and MAFFT / FFT-NS-i (Figure S1). In our phylogenetic reconstructions, the resolution at the level of the major lineages within the Narna-Levi clade was greater than at the level of the genera. The clustering of members of the mitovirus genera was not consistent among the three trees (Figure 2 and Figure S1). For example, the genus “Kvinmitovirus” nested within the genus *Triamitovirus* (Figure 2) or a small subclade of “arkeomitoviruses” branched within the formal mitoviruses clade (Figure S1). This apparent inconsistency between members of the mitovirus genera was clarified in subsequent reconstructions, as described in the Section 3.7, in which the monophyly of “Arkeomitovirinae”, formal mitoviruses, and mitoviruses genera were retained according to the ICTV report [20] and our previous study [21]. It should be remarked that the discrimination between mitoviruses, narnaviruses, narliviruses, botourmiaviruses and leviviruses shown in the present ML-trees (Figure 2 and Figure S1) was consistent with the work of Sadiq and colleagues [41].

The 144 curated sequences that fit into “Clade B” were found to be monophyletic and constituted a sister clade to formal mitoviruses, sharing a hypothetical recent common ancestor. In addition, the branch length of “Clade B” was shorter than the branch of the formal mitoviruses clade (*Mitoviridae*), suggesting a smaller number of evolutionary change events (0.1 and 0.5, respectively, of the estimated average number of substitutions per site) with respect to the hypothetical aa sequence of the most recent common ancestor of all mitoviruses. This branch length pattern was not reflected in the phylograms obtained by CLUSTAL-O and the FAFFT/FFT-NS-I alignment (Figure S1). The tree of the Figure 2 shows that new putative mitoviruses that accommodated in the “Clade A” (labeled with bold letters in the sub-tree corresponding to the formal mitoviruses) were found to be monophyletic, including OnuMV7 (genus *Kvaramitovirus*). The monophyly of “Clade A” was kept with high support in all the alignment methods employed in the present study (Figure S3). The globality of the phylogenetic analyses allows us to suggest the existence of a basally divergent lineage of putative mitoviruses, with respect to the mitoviruses formally recognized by the ICTV, and to be able to tentatively expand the genus *Kvaramitovirus*.

### 3.3. Proposal of Supra-Generic Taxa

The members of the basally divergent clade were manually inspected in order to evaluate the genetic architecture. Surprisingly, the deduced ORFs of the putative mitoviruses included in “Clade B” did not have an internal in-frame UGA. Hence, tryptophan (W) was encoded only by the UGG codon in all 144 members. Therefore, this is a structural feature that distinguishes the representative members of this new putative mitovirus lineage from the formal mitoviruses, as the latter present several internal UGA codons. Although the lack of UGA codons has rarely been reported in fungal mitoviruses [5], it is a feature of plant mitoviruses [6]. However, phylogenetic evidence has indicated that plant mitoviruses originated by a “horizontal jump” from a fungal mitovirus to form a monophyletic clade within the genus *Duamitovirus* [6]. Moreover, the UA content in the putative near-complete nt mitovirus genomes included in “Clade B” was, in general, lower than 60% (mean: 54.6%; SD: 4.1%), with this proportion being relatively low compared to formal mitoviruses, as these have a content greater than 55%, and more commonly of 60–70% (sequences details given in Table S4). These molecular characteristics of the putative mitoviral genomes are similar to those of the narnavirus genomes. In *Narnaviridae*, the relatively low proportion of AU and the absence of UGA codons within the ORF are apparently adaptations to replication in the host cell cytoplasm. So, in order to assess whether these viruses share genomic characteristics with narnaviruses, we scanned for the presence of long uninterrupted ORFs in the antisense strand (-ssRNA), a feature detected in narnaviruses (ambigrammatic genome) [46]. Although the putative mitoviral genomes included in “Clade B” cannot be considered as complete end-to-end sequences, there is no evidence for the existence of putative large reverse ORF (1000 nt or more) encoding of functional proteins. The sequences were manually scanned (Table S3) using the NCBI-ORFfinder [28] and the NCBI-CDS [29] programs. Finally, despite the fact that these viral sequences have an architecture that is more similar to narnaviruses than authentical mitoviruses, the absence of ambigrammatic sequences, the presence of the six aa sequence motifs typical of mitoviral RdRps and the monophyly (suggesting a recent common shared ancestry with the formal mitoviruses), seem to indicate that the “Clade B” belongs to the family *Mitoviridae*. The molecular characteristics of “arkeomitoviruses” shared with narnaviruses are probably the result of an evolutionary convergence caused by selection pressure from the cytoplasmic protein biosynthesis machinery.

According to our approaches, we believe that there is sufficient evidence to propose the existence of putative mitoviruses belonging to a basally divergent lineage, with this new lineage being relatively (evolutionary speaking) distant to the already proposed mitovirus genera. These results led us to propose a taxonomic division of the family *Mitoviridae* into two supra-generic taxa: (i) subfamily “Mitovirinae”, which involves the genera *Unuamitovirus*, *Duamitovirus*, *Triamitovirus* and *Kvaramitovirus*, and also the clade “Kvinmitovirus”; and (ii) subfamily “Arkeomitovirinae” (*arkeo* means ancient in the Esperanto language), which involves the putative mitoviruses initially included in “Clade B”.

### 3.4. The genus Kvaramitovirus: A Proposal for Expansion

The exemplar OnuMV7, belonging to the monospecific genus *Kvaramitovirus*, but with an unclear phylogenetic relationship [21], was robustly clustered with four new putative mitoviruses (“Clade A”) (Figure 2 and Figure S3). These putative mitoviruses were retrieved from the NCBI TSA database (GIFJ01468976.1) and the NCBI nr protein database (Grapevine-associated mitovirus 11, MW648458.1; Grapevine-associated mitovirus 12, MW648459.1 and Fusarium asiaticum mitovirus 8, MZ969058.1). The TSA sequence, identified by the NCBI accession number GIFJ01468976.1 and classified as a putative near-complete mitovirus genome in the present study, was obtained from a transcriptomic study of an exemplar of breadcrumb sponge (*Halichondria panacea*, *Porifera; Demospongiae*). This putative mitovirus branched at the root of the other four members and exhibited a greater number of cumulative evolutionary changes (Figure 2 and Figure S2). All these putative mitoviruses shared a high proportion of AU content: 67–73% (Table S4). The identity percentage among all combinations of pairwise alignments ranged from 24.00% to 47.00% (BLASTp alignment of protein sequences). This was lower than the species demarcation criterion (threshold of 70%) established by the ICTV Mitoviridae SG [20]. Thus, the protein sequence identity scores and the phylogenetic evidence reported here are strong reasons for proposing the following four new members, probably belonging to four new species within the genus *Kvaramitovirus*: “Halichondria panicea associated mitovirus 1” (GIFJ01468976.1)*,* Grapevine-associated mitovirus 11 (MW648458.1), Grapevine-associated mitovirus 12 (MW648459.1) and Fusarium asiaticum mitovirus 8 (MZ969058.1) (Table S2). Halichondria panicea associated mitovirus 1 was redeposited under the GenBank Third Party Annotation (TPA) accession number BK062826.1.

### 3.5. On the Origin of the New Putative Mitoviruses without In-Frame UGA(W) Codons

The putative near-complete mitovirus genomes included in the new proposed subfamily called “Arkeomitovirinae” were obtained from transcriptomic or metatranscriptomic studies from the whole or a part of the presumptive host (body, tissue, an organ, portion of the body, intestinal content, excrement or body fluids/exudates) and also from environmental samples. Although an RNA-seq comes from a single organism or an anatomical part of a single organism, it is not possible to discard its origin from a mitovirus-infected symbiont or a mitovirus-infected parasite. Studies on the dinucleotide frequency of the putative mitovirus genome and the presumptive host nuclear/mitochondrial genome should be performed for proper host assignment [8]. The host assignment for these new putative mitoviruses without in-frame UGA codons requires an in-depth analysis that will be addressed in a future study. It is also important to clarify that these sequences were identified from transcriptomic studies on organisms belonging to evolutionarily unrelated taxonomic groups, such as chlorophytes, phaeophytes, cnidarians, malacostracans, bivalves, gastropods, mammals, actinopterygians, poriferans, ascidiaceans, birds (Aves), insects and tunicates. In addition to these, several sequences were obtained from diverse metatranscriptome environments (ponds, lakes, rivers, paddy sediment), animals feces and land, among other sample sites. Thus, as these new putative mitoviruses without internal UGA codon sequences were generated from independent transcriptomic studies, cross-contamination events could be discarded. Interestingly, this grouping did not involve putative mitoviruses previously identified in fungi or plants. However, due to the impossibility of accessing the samples, it was not possible to determine whether these new putative mitoviruses without in-frame UGA codons came from a cytoplasmic / mitochondrial replicating mitovirus, or whether they originated from a transcriptionally active mitovirus sequence integrated into the host genome as a non-retroviral Endogenous RNA Viral Element (NERVE).

Recently, Jacquat and colleagues [21] noted the lack of concrete evidence about mitovirus integration into the genome of non-plant organisms. In our study, the putative mitovirus genome sequences included in “Clade B” lacked in-frame UGA within the ORFs, which could be indicative of an adaptation to a translation system that does not use UGA codons—for example, the Bacterial, Archaeal and Plant Plastid Code (T.T. n° 11), Chlorophycean Mitochondrial Code (T.T. n° 16), Scenedesmus obliquus Mitochondrial Code (T.T. n° 22), Thraustochytrium Mitochondrial Code (T.T. n° 23), or the nuclear/cytoplasmatic translation system of all eukaryotic cells. The wide distribution of the geographic origin of the sequences, as well as the great diversity of environments and organisms at these sample sites, could be indicative of putative mitoviruses that infect bacteria (the UGA codon is a signal of the termination of translation with no encoding of the tryptophan aa). This hypothesis is plausible since bacteria occupy every site as they are found in all habitats on Earth [47,48,49].

In order to explore this last possibility, two BLAST searches were performed. Firstly, we performed a tBLASTn search on bacteria (NCBI taxid:2) entries in the TSA database, using the fragment 199 aa of the RdRp coded by Sclerotinia sclerotiorum mitovirus 3 (the same as that used in the initial search in the present work) as the query. However, this search did not generate any hits for the 23 TSA databases registered (September 2022 update). Secondly, a similar search was performed on the NCBI–Whole Genome Shotgun (WGS) database to evaluate a possible mitovirus sequence integration into the bacterial chromosome (prokaryote Bacteria: NCBI taxid:2). Here, the only hit (NCBI accession: CACLOE010000022.1) did not reveal any significant similarity (33% of the query cover and an e-value of 0.006), and therefore its mitovirus origin was discarded. Finally, a bacterial origin of the putative codonless mitovirus UGA, included in the new proposed subfamily named “Arkeomitovirinae”, could be excluded. This conclusion is consistent with the extensive literature on bacterial viruses [50,51].

Fungi are considered to be the main hosts of mitoviruses, and hence, it might be expected that they are also the hosts of putative “arkeomitoviruses”. This idea is plausible since the fungi are part of the microbiota of almost all multicell organisms and environments in the biosphere [48,52,53]. Only a small group of fungi, mainly the most basally divergent fungi (non-ascomycetes and basidiomycetes fungi), do not use the UGA(W) in the mitochondrial genetic codes. In agreement with this, these fungi are infected by mitoviruses, with only a few or without any UGA codons in their ORFs in an adaptation process [5]. Although this hypothesis should be considered, the fungal origin of the putative mitoviruses belonging to “arkeomitoviruses” still remains to be demonstrated by experimental procedures.

An embryophyte origin was discarded because the mitoviruses that replicated in embryophytes were shown to be a monophyletic clade which evolutionarily originated from a horizontal jump from a fungal mitovirus belonging to the genus *Duamitovirus* [6]. Moreover, the origin of the NCBI biosamples rules out this possibility (Table S3).

Finally, to summarize, we do not have information to assign a host; however, the absence of internal UGA and the relatively low proportion of AU is consistent with an adaptation to the cytoplasmic/nuclear translation system of eukaryotic cells, regardless of their host genome integration or cytoplasmic autonomous replication.

### 3.6. A Coherent Picture of the Evolution of Mitoviruses

All the phylogenetic reconstructions performed here indicated that “Arkeomitovirinae” is a basally divergent lineage in the mitovirus evolutionary tree. The phylogenetic evidence and the distinctive structural features (no in-frame UGA and a low proportion of AU) led us to propose the existence of a new monophyletic clade of mitoviruses that could have had an impact on the known evolutionary scenario of mitoviruses.

**Figure 3:**
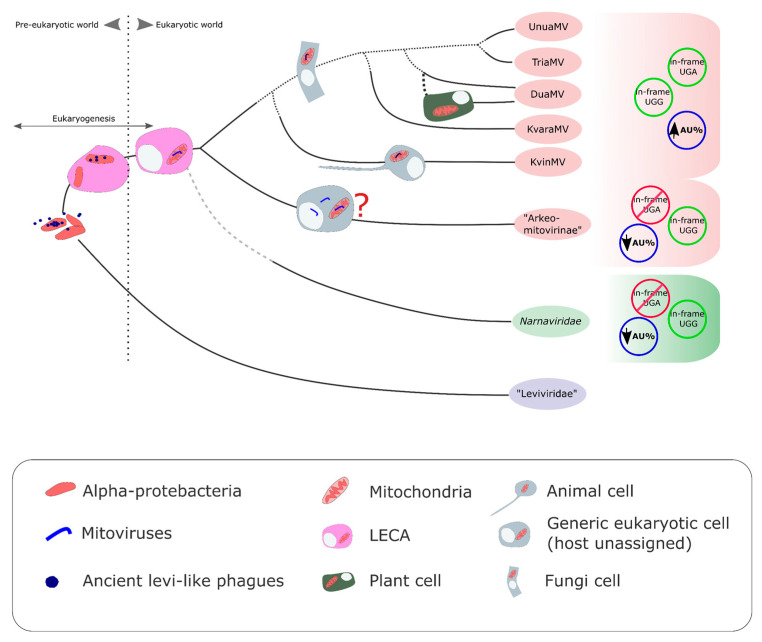
A comprehensive picture of the origin and evolutionary scenario of *Mitoviridae and Narnavridae*. The scheme has temporal directionality from left (past) to right (present). Lines represent the ancestor–descendant relationship of the main mitovirus lineages based on the results reported in the present work and those reported by Mitovirus SG [20], Nibert et al. [6] and Jacquat et al. [21]. Solid lines have phylogenetic support from protein sequences. Dotted lines do not have a good topological phylogenetic resolution. The green line indicates the evolutive origin of the monophyletic lineage of plant-replicating mitoviruses (blue string within the mitochondria) and endogenized plant mitoviruses (blue string within the nucleus). This lineage together with a lineage of mitoviruses that replicates in fungi (within mitochondria) constitute the genus *Duamitovirus* (DuaMV). Moreover, the genera *Unuamitovirus* (UnuaMV), *Triamitovirus* (TriaMV) and *Kvaramitovirus* (KvaraMV) are exclusively made up of fungal mitoviruses. “Kvinmitovirus” (KvinMV) is the recently proposed mitoviruses genus. These five clades are included in the proposed subfamily “Mitovirinae”, a sister clade of “Arkeomitovirinae”. There is no evidence concerning the authentic host of the putative “arkeomitoviruses”. The ancestor common to all mitoviruses was replicating within the mitochondria of the last eukaryotic common ancestor (LECA). This originated from levivirus-like bacteriophages that infected alpha-proteobacteria (mitochondrial precursors) in the pre-eukaryotic world. During eukaryogenesis, these levivirus-like phages became confined within the endosymbiont organelle (loss of lytic cycle) and evolved to become the ancestor of all mitoviruses by genome reduction. The main ideas of the originally proposed evolutive scenario detailed here were taken from Koonin and Dolja [54] and Koonin et al. [55].

**Figure 4:**
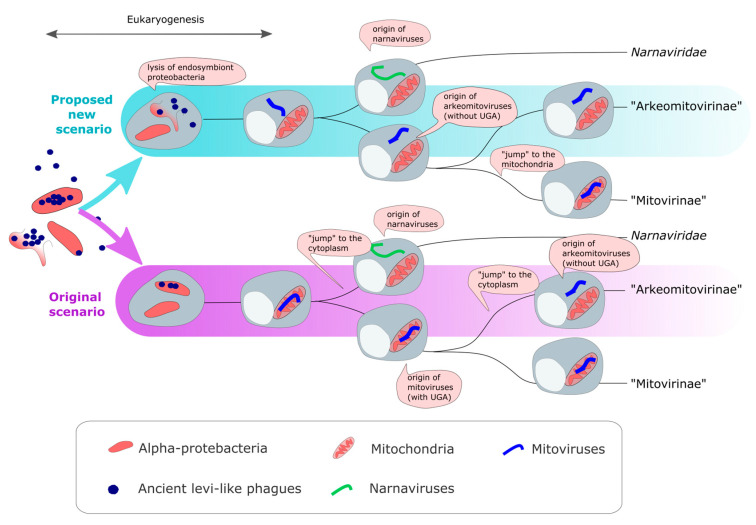
Currently accepted and proposed evolutionary scenarios of mitovirus origin. The scenarios are described in Section 3.5 and Section 3.6 of the paper.

The first ideas on the evolutionary scenario of mitoviruses were reported by Koonin and Dolja (2014) [54] and Koonin et al. (2015) [55] (Figure 3 and Figure 4). They proposed that ancient levivirus-like phages (inhabitants of the pre-eukaryotic world) infecting α-proteobacteria became “trapped” during eukaryogenesis. The loss (deletion) of genes associated with regressive evolution [56] may explain the transition from a multigenic (infectious levivirus-like virus) to a single gene (mito- and narna-like viruses) genome. In this way, these precursor viruses to existing mitovirus would lose their lytic capacity and become a naked replicon within the endosymbiotic organelle. In fact, this was demonstrated experimentally by Mills et al. (1967) [57], who found that an infectious virion-forming levivirus, phage Qβ, converted to a non-infectious non-capsid self-replicating ribonucleoprotein complex (mitovirus-like entity) [57]. Initially, it was thought that mitoviruses were exclusive to fungi [1,16]. Then, the discovery that all plant-replicating mitoviruses and plant-endogenous mitoviruses exhibit monophyly, and also probably originated from a horizontal jump of a glomeromycotan mitovirus, added another chapter to the evolutive scenario [6]. The evidence indicated that this was a unique evolutionary event. The ICTV Mitovirus SG classified the mitoviruses that replicate in plants within the genus *Duamitovirus*, a monophyletic clade consisting mainly of fungal mitoviruses. Another interesting finding probably expands the host range of mitoviruses beyond fungi and plants, including metazoans as the putative host [21], as evidence based on phylogenetic analysis indicated that fungal (genus *Unua-, Dua-, Tria-*, and *Kvaramitovirus*) and animal (Kvinmitovirus) mitoviruses probably share a recent common ancestor [21]. This hypothesis is consistent with the phylogeny of animals and fungi, which forms a monophyletic clade (Obazoa supergroup) [21]. This evolutionary scenario also proposed that current cytoplasmatic narnaviruses emerged from ancient mitovirus-like naked replicons by a “jump” to the cytoplasm (Figure 4). In light of new evidence, narnaviruses seemed to have evolved as a sister group to mitoviruses and gave rise to two other families of narna-like viruses: *Botourmiaviridae* and “Narliviridae” [41]. However, to simplify the discussion, we will only refer to the *Narnaviridae* family, which has evidence of sharing a common ancestor with mitoviruses.

In our present article, a basally divergent lineage is added. Although the information we recovered is not sufficient to assign hosts to these new putative mitoviruses without in-frame UGA codons, it is sufficiently relevant to be able to predict the existence of a lineage of mitoviruses with an unusual genetic architecture that emerged early in the evolutionary history of mitoviruses. In this way, two major current mitovirus lineages would have been configured, with internal UGA (“Mitovirinae”) and without internal UGA (“Arkeomitovirinae”). Although it is not possible to rule out their replication on host mitochondria or their endogenization into the host genome, the lack of in-frame UGA codons and the low proportion of AU in the genomes of the putative mitoviruses belonging to “Arkeomitovirinae” would seem to indicate their cytoplasmic location as being similar to that of narnaviruses, which have a low proportion of AU in their genomes and replicate in the cytoplasm [1]. However, this has to be demonstrated by experimental procedures.

### 3.7. An Alternative Parsimonious Scenario for the Origins of Mitoviruses

Although the authentic hosts and their replicative location of the “arkemitoviruses” remain to be determined, the lack of in-frame UGA codons and the low proportion of AU in the genomes would seem to indicate their cytoplasmic location as being similar to that of narnaviruses. In order to evaluate the evolutionary scenario of “arkemitoviruses” and formal mitoviruses, we analyzed the accumulated substitutions with respect to the most recent common ancestor of all mitoviruses. The phylogenic results obtained by PROMALS (designed to align divergent sequences) showed a smaller number of evolutionary change events for the “Arkeomitovirinae” clade. To corroborate this assumption, we made new phylogenetic inferences for members of the family *Mitoviridae* only. The sequences were aligned using different programs (PROMALS, CLUSTAL-O, MAFFT and EXPRESSO [58] ), and prior to building the trees, each alignment was used to estimate the best-fitting substitution model. The results are shown in Figure S3. The branch length of the “Arkeomitovirinae” clade was shorter than the branch of the “Mitovirinae” clade, suggesting a lower number of substitutions per alignment site: 0.5 vs 0.6, 0.1 vs. 0.2, 0.2 vs 0.4 for PROMALS, CLUSTAL-O and EXPRESSO derived ML-trees, respectively (Figure S3). The MAFFT-derived tree shows an inverse pattern. These differences are subtle, but it is worth noting that the evidence suggests directionality, indicating the “arkemitoviruses” to be a precursor to the mitochondrial mitoviruses. In this context, a new parsimonious scenario for the origins of *Mitoviridae* family is described below.

A levi-like phage ancestor that infected α-proteobacteria was released to the cytoplasm from a phagocytosed infected α-proteobacterium in the eukaryogenesis era. This release could have occurred by the lysis of the engulfed α-proteobacteria, with the consequent release to the host proto-eukaryotic cell cytoplasmic matrix of virions and viral components not assembled into virions (viral proteins and viral +ssRNA). This RNA viral genome of the ancestral levi-like phage could replicate in the cytoplasm since the prokaryotic genetic code (TT #11) of alpha-proteobacterium and the canonical eukaryotic genetic code (TT #1) are very similar (in both genetic codes the UGA codon is a translation stop signal). Viral RNAs would continue with replication in the cytoplasm of the first-born prokaryote, and the phages underwent genetic reduction up to becoming a non-infective cytoplasmic self-replicating entity, only maintaining the RNA replicase coding region. Under this proposed new scenario, the “arkeomitoviruses” of cytoplasmatic replication are the first forms of mitovirus to appear (see scheme in Figure 4). Then, the authentic mitochondrial replicating mitoviruses (“Mitovirinae”) are a sub-lineage that migrate to the mitochondria of the common ancestor of fungi and animals (obazoans). In this way, the two major current mitovirus lineages would have been configured to the sub-families “Mitovirinae” and “Arkeomitovirinae”.

The currently accepted evolutionary scenario posits that mitoviruses originated inside mitochondria (proto-mitochondria) from a levi-like phage ancestor “trapped” in the endosymbiotic organelle, and then, narnaviruses originated from a mitoviral member that jumped to the cytoplasm (Figure 4). Normally, the phages would have caused the rupture of the bacterial cell membrane (lysis). Why would it not have happened? The new parsimonious scenario for the origins of *Mitoviridae* family proposed here assumes that “arkeomitoviruses” of cytoplasmatic replication were the first forms of mitovirus to appear. The cytoplasmic origin was due to the release of levi-like phages, and the subsequent reductive evolution, into the cytoplasm caused by the “explosion” of an infected endosymbiont α-proteobacterium. That is, in this new scenario, it is proposed that the lysis would have occurred as expected. So, the hypothetical scenario (here proposed) posits that narnaviruses originated from a cytoplasmatic mitoviral ancestor. Then, a lineage of mitoviruses would have migrated into the mitochondria of the host lineage that gave rise to fungi and animals (Obazoa supergroup). In the mitochondrial environment, mitoviruses will have progressively gained internal UGA codons by selective pressure from the mitochondrial protein biosynthesis machinery (“Mitovirinae”). “Arkeomitoviruses” that remained in the cytoplasm did not gain internal UGA codons.

Phylogenetic reconstructions based on viral sequence present serious limitations for resolving deep phylogenies of small RNA viruses by their high mutation rates [59,60]. This feature explains the impossibility of establishing an unequivocal evolutionary directionality of the main lineages of the phylum *Lernaviricota* as discussed in Sadiq’s paper [41], especially between narnaviruses and mitoviruses. Therefore, the evolutionary scenarios proposed in this work are hypothetical and need to be confirmed.

## 4. Final Comments

In an article published in 2018, it was deduced that plant mitoviruses originated from a horizontal jump from a fungal host to the evolutionary precursor of today’s embryophytes. Then, another recently published work proposed the existence of a clade of putative mitoviruses that presumably replicated in animals. This in silico study was supported by previous evidence for the identification of a bona fide animal mitovirus that replicates in the mitochondria of a fly species. Here, the existence of a new monophyletic clade (“Arkeomitovirinae”) of putative mitoviruses that use the canonical genetic code to replicate (presumably), which would have an impact on the evolutionary scenario of the mitoviruses, is presented. Due to its apparent basal divergence into the *Mitoviridae* phylogenetic tree, and also its genetic signatures, the “Arkeomitovirinae” group has been provisionally placed in the taxonomic rank of subfamily. Our study can be supported by the study by Neri and colleagues [61] in which they predict the existence of a large number of putative mitoviruses that are possibly adapted to the eukaryotic nuclear genetic code [61].

In this paper, the expansion of the genus *Kvaramitovirus* is proposed, which only has one formal member so far. Additionally, finally, this is the first study that has produced a detailed description of the evolutionary history of mitoviruses, a group of viruses that have directly descended from the ancestral phages of the pre-eukaryotic world.

## Data Availability

The virus sequences analyzed in this study and retrieved from NCBI nr protein database are available at NCBI-GenBank under accession numbers detailed in the Supplementary Material section (Tables S1 and S3). The TSA sequences reported in the present study have been redeposited into the GenBank Third Party Annotation database of the NCBI under the following accession numbers and names: NCBI accession code: BK062826 (Halichondria panicea associated mitovirus 1); NCBI accession code BK062827 (Aplysina aerophoba associated mitovirus 1); NCBI accession code BK062828 (Ulva lactuca associated mitovirus 1); NCBI accession code BK062829 (Austropotamobius pallipes associated mitovirus 1); NCBI accession code BK062830 (Gigantidas vrijenhoeki associated mitovirus 1); NCBI accession code BK062831 (Psammomys obesus associated mitovirus 1) and NCBI accession code BK062832 (Polycarpa mytiligera associated mitovirus 1).

## Supplementary Materials

The following supporting information can be downloaded at https://www.mdpi.com/article/10.3390/v15020340/s1. Figure S1: Phylogenetic trees based on different alignments; Figure S2: High-resolution tree of Mitovirus RdRp amino acid sequences; Table S1: Details of sequences included in the phylogenetic reconstructions; Table S2: Result of the initial tBLASTn search of the NCBI TSA database and selection criteria; Table S3: Members of clades Kvaramitovirus and “Arkeomitovirinae”; Table S4: Molecular characteristics of mitoviruses and putative mitoviruses.
