# Supplementary material for: Putative Mitoviruses without In-Frame UGA(W) Codons: Evolutionary Implications
Source: Viruses. 2023 Jan 25;15(2):340. doi: 10.3390/v15020340 (PMC9965874; doi:10.3390/v15020340)
Supplement: Supplementary file 1 [file viruses-15-00340-s001.zip › Supplementary Figures.pdf]

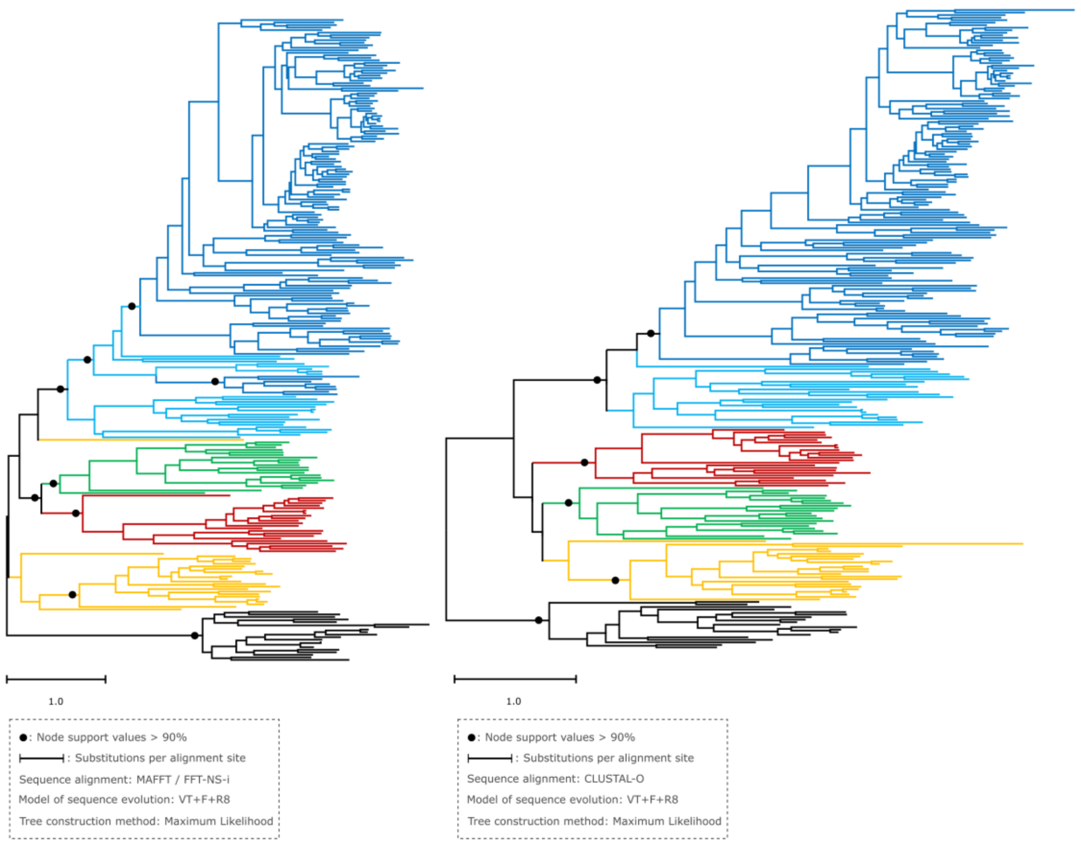

Figure S1. *Phylogenetic trees based on MAFFT and CLUSTAL-O alignments*. Generalized midpoint rooted tree of 254 RdRp aa sequences encoded by representative members of the phylum *Lenarviricota. Branches color-code: blue indicates the propose subfamily "Arkeomitovirinae" ("clade B"), cyan indicates "formal mitoviruses" fitted to the propose subfamily "Mitovirinae" (formal genera Unuamitovirus, Duamitovirus, Triamitovirus and Kvaramitovirus, and the proposed clade "Kvinmitovirus"). The family Mitoviridae comprises members of the blue and cyan branches. Orange, red and green indicate the families Narnaviridae, Botourmiaviridae and "Narliviridae". The supplementary Table S1 and S3 shows details of the 254 members. The phylogenetic reconstructions are detailed in the materials and methods section.*

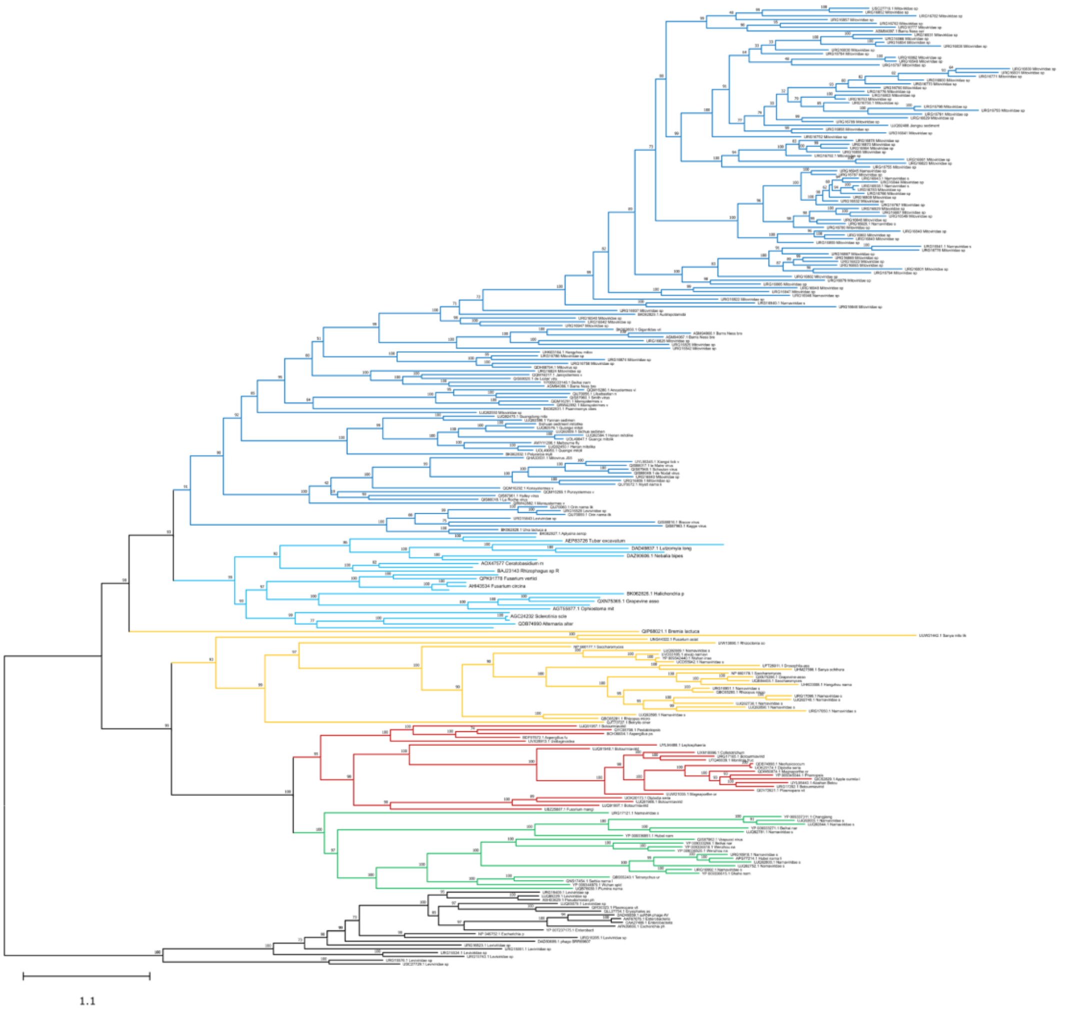

Figure S2. *High resolution tree of Mitovirus RdRp amino acid sequences based on PROMALS alignment*. Generalized midpoint rooted tree of 254 RdRp aa sequences encoded by representative members of the phylum *Lenarviricota. Branches color-code: blue indicates the propose subfamily "Arkeomitovirinae" ("clade B"), cyan indicates "formal mitoviruses" fitted to the propose subfamily "Mitovirinae" (formal genera Unuamitovirus, Duamitovirus, Triamitovirus and Kvaramitovirus, and the proposed clade "Kvinmitovirus"). The family Mitoviridae comprises members of the blue and cyan branches. Orange, red and green indicate the families Narnaviridae, Botourmiaviridae and "Narliviridae". The members are identified are labeled with the NCBI accession number and the virus names (Names were truncated to the first few characters). Tree construction method: Maximum Likelihood. Evolutive model: BLOSUM62+F+R7. Node support values are displayed as percentages. The bar indicates one substitution (estimated median number) per alignment site. The supplementary Table S1 and S3 shows details of the 254 members. The phylogenetic reconstructions are detailed in the materials and methods section.*

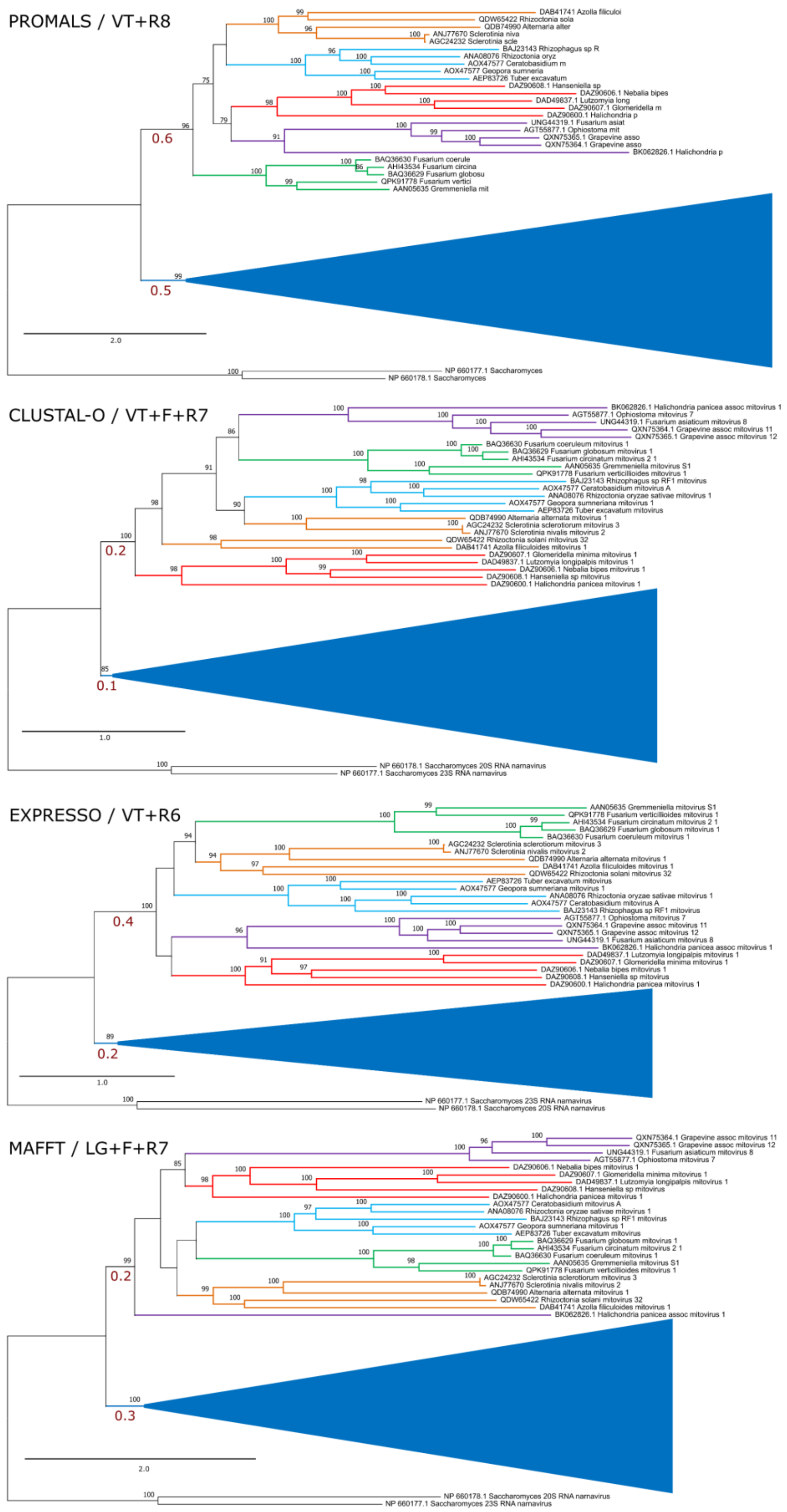

Figure S3. *Phylogenetic trees based on different alignments*. Maximum Likelihood trees of RNA replicase amino acid sequences based on PROMALS, CLUSTAL-O, EXPRESSO and MAFFT; and separated by a slash, the evolutionary

model that best fit to the respective alignment is indicated. Color code: purple indicates “clade A” (genus *Kvaramitovirus*) with the new putative members included in this work and the collapsed blue clade indicates clade “B” (“Arkeomitovirinae”) formed by 144 exemplars. Red branches indicate member of the recently propose genus *kvinmitovirus* an green, orange and cyan indicates member of genus *Unuamitovirus, Duamitovirus and Triamitovirus*, respectively. Below the branches of the two major clades, the estimated average substitutions per alignment site are shown in dark red. Node support values were obtained according to the UFBoot2 (1000 pseudo-replicates) test and are displayed as percentages. Root tree: generalized midpoint optimization. The bar indicates the number of substitutions (estimated median number) per alignment site
